# Prediction of CD8^+^ Epitopes in *Leishmania braziliensis* Proteins Using EPIBOT: *In Silico* Search and *In Vivo* Validation

**DOI:** 10.1371/journal.pone.0124786

**Published:** 2015-04-23

**Authors:** Angelo Duarte, Artur T. L. Queiroz, Rafael Tosta, Augusto M. Carvalho, Carlos Henrique Barbosa, Maria Bellio, Camila I. de Oliveira, Manoel Barral-Netto

**Affiliations:** 1 Departmento de Tecnologia, Universidade Estadual de Feira de Santana, Av. Transnordestina, s/n, DTEC-Módulo 3, 44036–900, Feira de Santana, BA, Brazil; 2 CPqGM—FIOCRUZ, R. Waldemar Falcão, 121, 40296–710, Salvador, BA, Brazil; 3 Instituto de Microbiologia Paulo de Góes, Centro de Ciências da Saúde, Universidade Federal do Rio de Janeiro (UFRJ), Avenida Carlos Chagas Filho, 373 Bloco D, sala 35, Cidade Universitária, 21941–902, Rio de Janeiro, RJ, Brazil; 4 Instituto de Investigação em Imunologia, São Paulo, Brazil; INRS - Institut Armand Frappier, CANADA

## Abstract

**Background:**

Leishmaniasis is caused by intracellular *Leishmania* parasites that induce a T-cell mediated response associated with recognition of CD4^+^ and CD8^+^ T cell Line 1Lineepitopes. Identification of CD8^+^ antigenic determinants is crucial for vaccine and therapy development. Herein, we developed an open-source software dedicated to search and compile data obtained from currently available on line prediction algorithms.

**Methodology/Principal Findings:**

We developed a two-phase algorithm and implemented in an open source software called EPIBOT, that consolidates the results obtained with single prediction algorithms, generating a final output in which epitopes are ranked. EPIBOT was initially trained using a set of 831 known epitopes from 397 proteins from IEDB. We then screened 63 *Leishmania braziliensis* vaccine candidates with the EPIBOT trained tool to search for CD8^+^ T cell epitopes. A proof-of-concept experiment was conducted with the top eight CD8^+^ epitopes, elected by EPIBOT. To do this, the elected peptides were synthesized and validated for their *in vivo* cytotoxicity. Among the tested epitopes, three were able to induce lysis of pulsed-target cells.

**Conclusion:**

Our results show that EPIBOT can successfully search across existing prediction tools, generating a compiled list of candidate CD8^+^ epitopes. This software is fast and a simple search engine that can be customized to search over different MHC alleles or HLA haplotypes.

## Introduction

Leishmaniasis is an infectious disease with significant economic impact in several countries. Over three hundred million people are exposed to the parasites, with 12 million infected worldwide, predominantly in tropical and subtropical countries (World Health Organization page: http://www.who.int/emc/diseases/leish/leisdis1.html). Leishmaniasis can be caused by different species of *Leishmania spp*. protozoans that infect macrophages in the human host. The treatments available for all forms of leishmaniasis are toxic and drug resistance is on the rise, further increasing the need for vaccine development [1].

In Brazil, cutaneous leishmaniasis (CL) is caused mostly by *Leishmania braziliensis* and presents as a skin ulcer associated with an intense inflammatory reaction with the presence of T cells [2]. The main immune mechanism for the control of *Leishmania* infection is IFN-γ production and subsequent macrophage activation, enabling the elimination of intracellular *Leishmania* parasites. It has been shown that CD4^+^ T cells are the main source of IFN- γ production, and, hence, macrophage activation (rev. in [3]). CD8^+^ T cells, on the other hand, are cytotoxic and contribute with pathogenesis of CL caused by *L*. *braziliensis*: CD8^+^ T cells promote lysis of leishmania-infected cells [4,5]. Moreover, the frequency of CD8^+^ T cells expressing cytotoxic mediators such as granzyme is directly correlated with the intensity of inflammatory reaction CL lesions [6]. The activation of CD8^+^ T cells is dependent on the recognition of antigenic peptide, presented by major histocompatibility complex molecules at the surface of infected cells [7]. Given the association of CD8^+^ T cell with pathogenesis of CL, it has become important to identify putative *L*. *braziliensis* CD8^+^ peptides associated with the cytotoxic response.

Experimental identification of MHC-binding peptides requires an assay for each peptide, a time consuming and costly process [8]. *In silico* epitope and MHC-peptide binding prediction, on the other hand, allow optimization of epitope discovery in vaccine design studies, therefore reducing the experimental workload [9]. A variety of algorithms have been developed and used in the field of epitope prediction and these algorithms range from Simplest Sequence Motifs and position-specific scoring matrices (PSSM) [10] to more complex machine-learning probabilistic approaches, such as Hidden Markov Models (HMM) [11], Artificial Neural Networks (ANN) [12] and Support Vector Machines (SVM) [13]. However, epitope discovery using distinct algorithms results in contrasting outputs, rendering candidate selection a cumbersome task. Since epitope mapping is important for the screening of cellular immunity in protected individuals, for example, an algorithm than combines different search methodologies and generates a unique list of candidates becomes a useful tool.

Here, we developed a two-phase algorithm that merges the results generated by individual prediction algorithms generating a unified final rank of elected epitopes. The algorithm was implemented in EPIBOT, a free software for non-commercial use developed in JAVA language. The zip source is available at: https://sites.google.com/a/ecomp.uefs.br/angeloduarte/epibot?pli=1. To run the friendly-interface, the user needs to extract the EPIBOT.rar file and navigate to folder. To execute in Windows and Mac OS, user double clicks the. jar file. For Linux OS execute the shell command java—jar Epibot.jar. More information is available in the software manual. EPIBOT was tested with 63 *Leishmania braziliensis* proteins and the top predicted eight epitopes were validated *in vivo* for their potential to induce CD8^+^ T cell cytotoxicity.

## Materials and Methods

### Software development

EPIBOT callibration and training. To measure the accuracy of the prediction tools (netMHC, SYFPEITHII, BIMAS, SVMHC and IEDB), we initially provided EPIBOT with a dataset of proteins containing known CD8^+^ epitopes: we used a set of 4251 H2-K^d^ epitopes, available in IEDB. However, among these only 831 proteins (S1 Table) with CD8^+^ epitopes were recovered from NCBI. Entries without reports at duplicates and proteins with more than 1 epitope were also removed from the dataset leaving 397 proteins (S2 Table) with identified CD8^+^ epitopes. These were used to assess the accuracy of the different prediction algorithms. Each calibration protein was then individually submitted to the following epitope prediction algorithm: BIMAS [14], SYFPEITHII [10], netMHC [15], SVMHC [16] and IEDB In every case, we searched for peptides presenting high affinity with mouse MHC class I molecules of BALB/c mice (H2- K^d^). Each search resulted in an epitope rank, where the best ranked epitope is expected to present a top score (rank = 1). This list was ordered according to the peptide score and the top epitopes ranked equal or close to 1. Therefore, each epitope rank (its compiled list position) is the algorithm´s prediction quality indicator. A perfect algorithm would classify known CD8 epitopes at the top. Thus, a Specific Prediction Score (SPS) was defined: this scores the algorithm’s quality for a specific calibration protein (and its calibration epitope). The SPS is the inverse of the Rank of the Calibration Epitope (RCE), from the list of epitopes yielded by the algorithm, presented in Eq 1:

SPS=1/RCE (1)

To assess overall algorithm quality, a General Prediction Score (GPS) was determined for each prediction algorithm, using all proteins SPS averages measured from the calibration set (defined in Eq 2). The Number of Calibration Proteins (NCP) was applied. The factor 10 was used to normalize GPS range to 0–10 interval. Good prediction algorithms are expected to have a GPS near 10.SPS

$$GPS=\frac{10}{NCP}\;\sum_{i=1}^{NCP}GPS_{i}$$ (2)

After calibration phase, the software determines the GPS value for each prediction algorithm. Thus, the EPIBOT algorithm is trained and ready to perform epitope screening in the query protein set.

### EPIBOT Unified prediction

After EPIBOT calibration, the GPS values are used to predict epitopes in a query dataset. Thereby, the two-step consolidation algorithm submits each query protein individually to the prediction algorithms (netMHC, SYFPEITHII, BIMAS, SVMHC and IEDB) and then measures the Unified Epitope Score (UES). First, the epitope-predicted list generated by each algorithm was normalized, where normalized scores (NS) range from [0–1] where 1 is the top epitope score and 0 is the bottom epitope score. Second, for a given Number of Prediction Algorithms (NPA), the UES is calculated using Eq 3, by summing epitope normalized scores, from each prediction algorithm, multiplied by the general prediction score from each different algorithm.

$$UES=\sum_{i-1}^{NPA}NS_{i}\times GPS_{i}$$ (3)

The final EPIBOT output is an epitope-predicted list descending by UES order, based on prediction from a prediction algorithm set defined by the user. Thus, the top epitopes are the best potential epitopes.

### EPIBOT prediction using *L*. *braziliensis* query proteins

Sixty-three *Leishmania braziliensis* proteins were screened with EPIBOT for the discovery of potential 9-mer epitopes presented by H2-K^d^ (Table 1). These epitopes were then validated in vivo, using a cytotoxicity assay, as described below. The full sequences of the proteins were extracted from NCBI. The selected proteins have been previously defined as candidate antigens [17–20] and were tested with the trained EPIBOT algorithm, using the five prediction tools mentioned above. The prediction output was evaluated and sorted by UES. The top epitopes were compared to mouse proteins using BLAST [11].Epitopes with query coverage of 100% and identity superior to 90% with self-murine proteins from ref_seq database were discarded. The predicted peptides were also compared to known epitopes from IEDB database to avoid known-epitopes re-evaluation.

**Table 1:** *L*. *braziliensis* protein query set.

Accession Number	Description	Accession Number	Description
XP001562139	cathepsin L-like protease	XP001562925	GP63, leishmanolysin
XP001562140	cathepsin L-like protease	XP001562927	GP63, leishmanolysin
XP001562141	cathepsin L-like protease	XP001562928	GP63, leishmanolysin
XP001562145	stress-induced protein sti1	XP001562929	GP63, leishmanolysin
XP001562173	histone H4	XP001562930	GP63, leishmanolysin
XP001562184	histone H4	XP001562931	GP63, leishmanolysin
XP001562231	histone H4	XP001562932	GP63, leishmanolysin
XP001568634	histone H4	XP001562933	GP63, leishmanolysin
XP001561520	histone H4	XP001562934	GP63, leishmanolysin
XP001562231	histone H4	XP001562935	GP63, leishmanolysin
XP001562753	histone H2B	XP001562936	GP63, leishmanolysin
XP001562816	GP63, leishmanolysin	XP001562937	GP63, leishmanolysin
XP001562817	GP63, leishmanolysin	XP001562938	GP63, leishmanolysin
XP001562818	GP63, leishmanolysin	XP001563556	tryparedoxin peroxidase
XP001562819	GP63, leishmanolysin	XP001563966	UDP-galactose transporter
XP001562820	GP63, leishmanolysin	XP001564130	histone H2B
XP001562821	GP63, leishmanolysin	XP001564131	histone H2B
XP001562822	GP63, leishmanolysin	XP001564132	histone H2B
XP001562823	GP63, leishmanolysin	XP001564191	putative histone H3 variant
XP001562824	GP63, leishmanolysin	XP001564262	cysteine peptidase A (CPA)
XP001562825	GP63, leishmanolysin	XP001564551	lipophosphoglycan biosynthetic protein (lpg2)
XP001562826	GP63, leishmanolysin	XP001564819	histone H2A
XP001562827	GP63, leishmanolysin	XP001565254	histone H4
XP001562827	GP63, leishmanolysin	XP001566056	histone H2B variant
XP001562828	GP63, leishmanolysin	XP001566321	activated protein kinase c receptor (LACK)
XP001562829	GP63, leishmanolysin	XP001566431	cysteine peptidase C (CPC)
XP001562830	GP63, leishmanolysin	XP001567803	heat shock protein 83–1
XP001562865	histone H3	XP001567804	heat shock protein 83–1
XP001562920	GP63, leishmanolysin	XP001568323	kinetoplastid membrane protein-11
XP001562921	GP63, leishmanolysin	XP001568634	histone H4
XP001562923	GP63, leishmanolysin		

### *In vivo* validation of predicted epitopes

#### Ethics Statement

Female BALB/c mice, 6–8 weeks of age, 25–30 grams, were obtained from CPqGM/FIOCRUZ animal facility where they were maintained under pathogen-free conditions. Mice were housed in groups of five. Environmental conditions were a temperature of 21°C ±2°, humidity of 55% ±10%, and a 12:12 light:dark cycle with lights on at 0700 and off at 1900. Animals were housed in 422×230×203 mm cages (Domi Series, Alesco, Brazil) and given access to mouse maintenance food (Biobase Bio Tec, Brazil) and water *ad libitum*. Environmental enrichment included bedding (maravalha *Pinus elliotti*, Hemo In Produtos para Biotério, Brazil), one red tinted igloo (Alesco, Brazil). During housing, animals were monitored twice daily for health status. No adverse events were observed. In vivo validation was performed using 36 animals. Splenocytes to be pulsed with peptides were obtained from 12 naive mice. Pulsed and labeled splenocytes were injected into 24 *L*. *braziliensis*-infected mice. All animal work was conducted according to the Guidelines for Animal Experimentation of the Colégio Brasileiro de Experimentação Animal and of the Conselho Nacional de Controle de Experimentação Animal. The local Ethics Committee on Animal Care and Utilization (CEUA) approved all procedures involving animals (L-03/2011). All sections of this report adhere to the ARRIVE Guidelines for reporting animal research. A completed ARRIVE guidelines checklist is included in S1 ARRIVE Checklist. To obtain splenocytes and draining lymph node cells, mice were euthanized using compressed CO_2_ under a flow rate of 1–3 liters per minute for a 10 liter (volume) chamber. CO_2_ flow was maintained for a minimum of 1 minute until lack of respiration and faded eye color were observed.

#### Parasite culture, intradermal inoculation and lesion measurement

*L*. *braziliensis* promastigotes strain MHOM/BR/01/BA788 were grown in Schneider medium (Sigma) supplemented with 100U/ml of penicillin, 100ug/ml of streptomycin, 10% heat-inactivated fetal calf serum (all from Life Technologies). Stationary-phase promastigotes (10^5^ parasites in 10μl of saline) were inoculated into the right ear dermis of age-matched BALB/c mice using a 27.5-gauge needle. Lesion size was monitored weekly, for 5 weeks, using a digital calliper (Thomas Scientific).

#### *In vivo* cytotoxicity assay

Splenocytes were obtained from naive BALB/c (H2-Kd). Cells were divided into three groups and were labeled with the fluorogenic dye CFDA (Invitrogen) at final concentrations of 8 μM (CFDA^high^), 2 μM (CFDA^intermediate^) or 0.5 μM (CFDA^low^). CFDA^high^ cells were previously pulsed for 40 minutes at 37°C with 5 μM of H-2K^d^-restricted *L*. *braziliensis* peptides (AYLASCDFI, AYIDGHVTI, KYQHSTEML or TYQRVYATL. CFDA^intermediate^ cells were pulsed in the same way with WYLATHSLI, SYMGYFQNI, IYVSYADLI or VYLSFGFRL). CFDA^low^ cells remained unpulsed. Subsequently, CFDA^high^ and CFDA^intermediate^ cells were washed and mixed with equal numbers of CFDA^low^ cells before injecting intravenously (15–20 x 10^6^ total cells) into *L*. *braziliensis*-infected and into control non-infected mice. Draining lymph node and spleen cells of recipient mice were collected 20 hours after cell transfer, fixed and analyzed by cytometry, using a FACSCalibur Cytometer (BD Biosciences). Percentage of CFDA^low^ (M1), CFDA^intermediate^ (M2) and CFDA^high^ (M3) cells were obtained using CellQuest software (BD Biosciences). Percentage of specific lysis was determined using the formula: 1 –((M2 infected/M1 infected) / (M2 naïve/ M1 naïve)) x 100% or 1 –((M3 infected/M1 infected) / (M3 naïve/ M1 naïve)) x 100% [21].

### Statistical analysis

A non-parametrical Mann Whitney U test was used to evaluate the difference between groups. *P*value less than 0.05 have been considered significant.

## Results

### *In silico* analysis of *L*. *braziliensis* proteins for potential candidate peptides

To identify potential CD8^+^ T cell activating 9-mer epitopes presented by H2-K^d^ we used the trained EPIBOT on a query dataset of 63 *L*. *braziliensis* protein candidates (Table 1). EPIBOT screening generated an output of 10950 UES-associated epitopes. The output list was sorted and the eight best-predicted epitopes with UES above 9.5 (Table 2) were chosen: AYLASCDFI, WYLATHSLI, AYIDGHVTI, SYMGYFQNI, GYVGIVVAL, KYQHSTEML, IYVSYADLI, VYLSFGFRL. These peptides were synthesized and validated *in vivo*.

**Table 2:** Top *L*. *braziliensis* epitopes as predicted by EPIBOT.

EPITOPE	UES^a^	Accession	Description	% Lysis (mean of spleen and dLN values)
AYLASCDFI	13.296	XP001562821	GP63 leishmanolysin	5.75
AYLASCDFL	13.193	XP001562826	GP63 leishmanolysin	NT^b^
AYLATCDFL	13.003	XP001562925	GP63 leishmanolysin	NT
WYLATHSLI	12.534	XP001562139	cathepsin L-like protease(CPB)	44.88
AYIDGHVTI	12.063	XP001562141	cathepsin L-like protease(CPB)	43.82
SYMGYFQNI	11.183	XP001564262	cysteine peptidase A (CPA)	8.28
GYVGIVVAL	10.56	XP001564551	lipophosphoglycan biosynthetic protein (lpg2)	NT
KYQHSTEML	10.505	XP001564191	histone H3 variant	12.02
IYVSYADLI	9.979	XP001562817	GP63 leishmanolysin	54.17
TYQRVYATL	9.947	XP001562141	cathepsin L-like protease(CPB)	7.15
VYLSFGFRL	9.601	XP001562184	histone H4	9.91

^a^UES, Unified Epitope Score

^b^NT: not tested

### *In vivo* cytotoxicity following inoculation of peptide-pulsed splenocytes into *L*. *braziliensis*-infected mice

**Fig 1:**
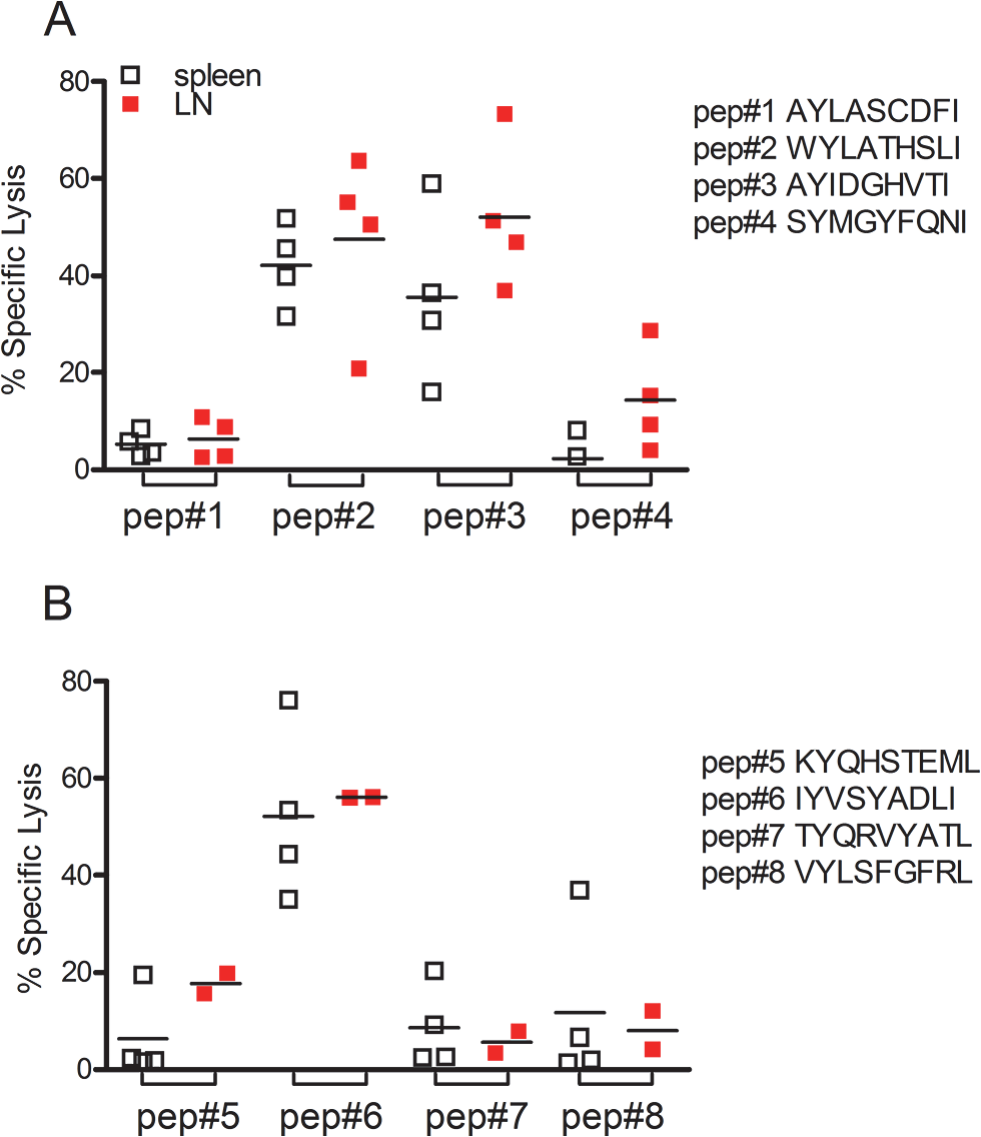
Specific CD8^+^ T cell-mediated immune response in *L*. *braziliensis*-infected BALB/c mice. Target cells were pulsed with *L*. *braziliensis* peptides (A) AYLASCDFI, WYLATHSLI, AYIDGHVTI and SYMGYFQNI and (B) GYVGIVVAL, KYQHSTEML, IYVSYADLY and VYLSFGFRL. Target cells were then transferred to *L*. *braziliensis* infected mice. In vivo cytoxoxicity was analyzed in spleen (white squares) and in draining lymph nodes (red squares) of recipient mice, by flow cytometry, as described in Materials and Methods. Data are shown individually.

Following epitope prediction using EpiBot, we validated the top eight peptides *in vivo*, using an assay that measures cytotoxicity of CD8^+^ T cells following an encounter with peptide-pulsed cells. No expressive cytotoxicity activity was observed when cells were pulsed with AYLATCDFI or SYMGYFQNI peptides (Fig 1A). On the other hand, the percentage of specific lysis of cells pulsed with WYLATHSLI was 42.2% in the spleen and 47.6% in the draining lymph node, while specific lysis for cells pulsed with AYIDGHVTI was 35.5%, in the spleen and and 52.1% % in the draining lymph node. Among the other four peptides tested, specific lysis was found only for peptide IYVSYADLI which displayed 52% and 56% of specif lysis in the spleen and draining lymph nodes, respectively (Fig 1B).

## Discussion

The identification of MHC-associated epitopes, recognized by T cells, is essential to measure epitope-specific T cell responses. Several approaches have been developed to improve epitope discovery (rev. in [9]), however, the variability observed among existing prediction tools rises important questions concerning the approaches´ consensus. Herein, we developed an analysis tool, EPIBOT, capable of epitope prediction across different prediction algorithms currently available online. EPIBOT was tested on query set of 63 *L*. *braziliensis* and the top eight epitopes were synthesized for in vivo validation.

Although the use of EPIBOT on 63 proteins from the *L*. *braziliensis* proteome yielded a large number of predicted epitopes, such result was lower than that obtained using different prediction tool (BIMAS, SYFPEITHII, netMHC, SVMHC and IEDB) individually.

After calibration, EPIBOT automatically submits each protein to the prediction tools selected by the user and all outputs are stored in an SQL database. The user only defines the protein queries set in a single initial submission. This solves all multi-submissions and high throughput issues. These features allow the user to test different prediction tools in large queries, containing many different proteins, thereby improving epitope search. In this way, EPIBOT handles large data sets, generated following the search by each predictor, combining different methodologies together with a rational output, yielding a unified score.

EPIBOT was able to handle the data groups generated by each predictor, combining different methodologies together with a rational output and generating a unique predictor score. The approach of combining results from several prediction tools is advantageous since integration of many prediction methods improves the overall prediction performance [22]. Additionally, this helps solving a crucial issue regarding different epitope prediction algorithms: the different score metrics used by each algorithm. For example, BIMAS [14] uses HLA class I half time dissociation, while SYFPEITHI [10] uses log-based score. Moreover, EPIBOT enables the use of different predictors tools in a set of proteins, generating a predicted epitopes pool, allowing epitope comparison based on a single score. This poses advantages over the use of a single prediction algorithm at a time so that the user can submit a query dataset at once instead of submitting each protein to each algorithm, one at a time. Besides, EPIBOT generates a simpler rational output in which the resulting epitopes are already ranked. Lastly, we compared the output of EPIBOT with that of some existing algorithms, using the same set of proteins tested in two published studies: Seyed et al. [23] described the results of *in-silico* prediction for six *L*. *major* proteins and indicated 18 epitopes for the HLA-0201 allele. Using the same set of proteins, EPIBOT identified 10 of the 18 epitopes in the top 10 positions (of the output list) and 15 of the 18 epitopes in the top 19 positions (of the output list). Agallou et al. [24] evaluated four leishmania proteins: 18 epitopes were predicted by SYFPEITHI and 24 epitopes predicted Bimas for the H2-K^d^ allele. Bimas did not predict certain epitopes predicted by SYFPEITHI and vice-versa; only 12 epitopes were predicted by both algorithms. Using this same set of proteins, EPIBOT yielded a result list in which seven of these 12 epitopes were in the top 10 positions, one epitope was in the 59^th^ position and the remaining four epitopes above the position 200. These results confirmed that EPIBOT performs well and is capable of combining the results of different prediction sites, yielding a more robust and meaningful prediction.

Another crucial issue generally observed across epitope prediction tools is the setting of output threshold. The choice of the threshold remains unclear and it is generally decided upon by the user: in one work, authors chose 20 as the threshold score for SYFPEITHI and 100 for BIMAS for the identification of *L*. *major* epitopes [23]. Elsewhere, the output threshold was defined by the top 5 highest scoring epitopes, among multiple predictors used [25]. Threshold cut-offs were also applied for epitope discovery in *L*. *infantum*. However, a score ≥18 for SYFPEITHI and binding affinity <500 nM for NetMCH were used for merging queries´ search [24]. EPIBOT, on the other hand, executes a rational comparison of the outputs from several prediction tools, which employ different prediction metrics.

Using EPIBOT with a query set of 63 *L*. *braziliensis* proteins, the software identified 10,950 *L*. *braziliensis* peptides. We then opted to validate the top eight predicted epitopes, *in vivo* as targets for CD8^+^ T cells. In *T*. *cruzi* infection, a similar assay detected >90% specific lyses in infected mice when cells were pulsed with the *T*. *cruzi* peptide VNHRFTLV [26]. The epitopes validated *in vivo* derived from *L*. *braziliensis* cathepsin L-like protein and Histone H3 [27]. Different studies showed that CD8^+^ T cells contribute with immunity to leishmania [28–31]. In the case of *L*. *braziliensis* infection, however, mice depleted of CD8^+^ T cells develop smaller lesions when compared with mice treated with isotype control antibody [5]. In addition, transfer of CD8^+^ T cells to Rag^–/–^ mice, that lack T cells, infected with *L*. *braziliensis* results in the development of uncontrolled and metastatic lesions. Nonetheless, CD8^+^ T cells producing IFN-γ are also detected in *L*. *braziliensis*-infected mice [32], indicating that these cells can contribute with macrophage activation and parasite elimination. Therefore, EPIBOT will contribute towards the identification of CD8 epitopes, enabling a better understanding of the role of CD8 cells during leishmania infection. Of note, comparing EPIBOT, BIMAS, SYFPEITHII, IEDB, NetMHC and SVMHC, only BIMAS was able to identify, among the 63 *L*. *braziliensis* proteins, the same three epitopes we herein validated in vivo. The other predictors, when used alone, identified either one or two epitopes only (data not shown).

Our results are in line with reports in the literature indicating the feasibility of using *in silico* predictors to identify CD8^+^ epitopes within *Leishmania* proteins [23,25]. However, we demonstrate that a combined approach such as EPIBOT accelerates epitope discovery in the sense that predictions are compiled from different algorithms. Indeed, among the eight epitopes tested, three induced cytotoxic activity *in vivo*, validating our search mechanism. Importantly, it was recently shown that the combination of epitope predictors, such as B cells predictors, yielded better results with protozoan parasite proteins [33].

## Supporting Information

S1 Arrive Checklist (DOCX)

S1 Table List of previously described CD8^+^ IEDB epitopes used to train EPIBOT. (DOCX)

S2 Table List of proteins with known CD8^+^ epitopes used to train EPIBOT. (DOCX)
