# Correction: Prediction of CD8^+^ Epitopes in *Leishmania braziliensis* Proteins Using EPIBOT: *In Silico* Search and *In Vivo* Validation

**DOI:** 10.1371/journal.pone.0129475

**Published:** 2015-06-01

**Authors:** Angelo Duarte, Artur T. L. Queiroz, Rafael Tosta, Augusto M. Carvalho, Carlos Henrique Barbosa, Maria Bellio, Camila I. de Oliveira, Manoel Barral-Netto

There are grant numbers missing from the Funding section. The correct funding information is as follows: This work was supported with grants from FAPESB (Fundação de Amparo à Pesquisa da Bahia) (SUS0003/2009, DCR002/2011 and RED0018/2013). The funders had no role in study design, data collection and analysis, decision to publish, or preparation of the manuscript.
