# Supplementary material for: Prediction of CD8+ Epitopes in Leishmania braziliensis Proteins Using EPIBOT: In Silico Search and In Vivo Validation
Source: PLoS One. 2015 Apr 23;10(4):e0124786. doi: 10.1371/journal.pone.0124786 (PMC4407964; doi:10.1371/journal.pone.0124786)

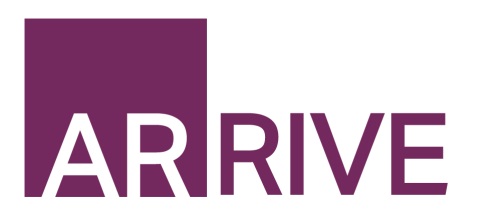


The ARRIVE Guidelines Checklist

Animal Research: Reporting In Vivo Experiments

Carol Kilkenny^1^, William J Browne^2^, Innes C Cuthill^3^, Michael Emerson^4^ and Douglas G Altman^5^

*^1^The National Centre for the Replacement, Refinement and Reduction of Animals in Research, London, UK, ^2^School of Veterinary Science, University of Bristol, Bristol, UK, ^3^School of Biological Sciences, University of Bristol, Bristol, UK, ^4^National Heart and Lung Institute, Imperial College London, UK, ^5^Centre for Statistics in Medicine, University of Oxford, Oxford, UK.*

|  | | ITEM | RECOMMENDATION | Section/ Paragraph |
| --- | --- | --- | --- | --- |
| 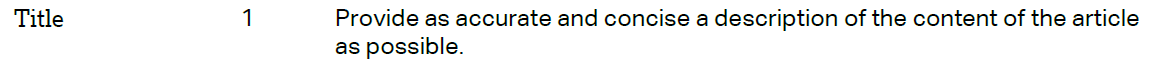 | | | Title, Line |  |
| 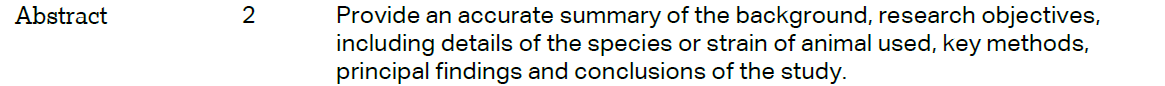 | | | Abstract, |  |
| INTRODUCTION | | |  |  |
| 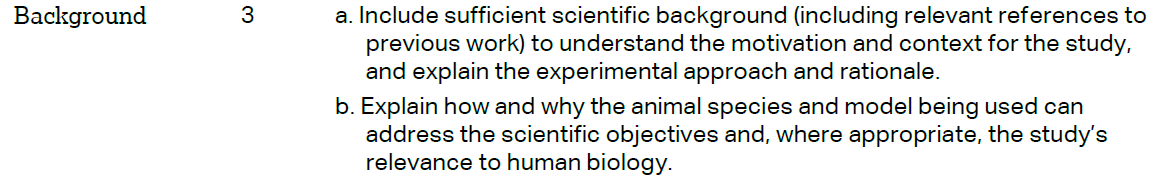 | | | Introduction, paragraphs #1m, #2 and #3 |  |
| 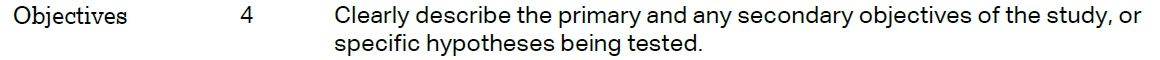 | | | Introduction, paragrah #4 |  |
| METHODS | | |  |  |
| 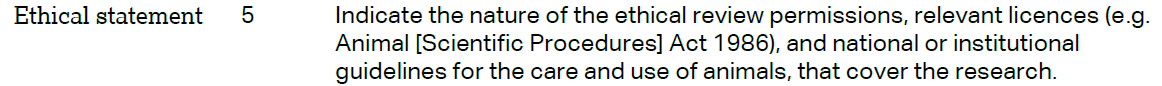 | | | Materials and Methods, In vivo validation, Paragrah #1 |  |
| 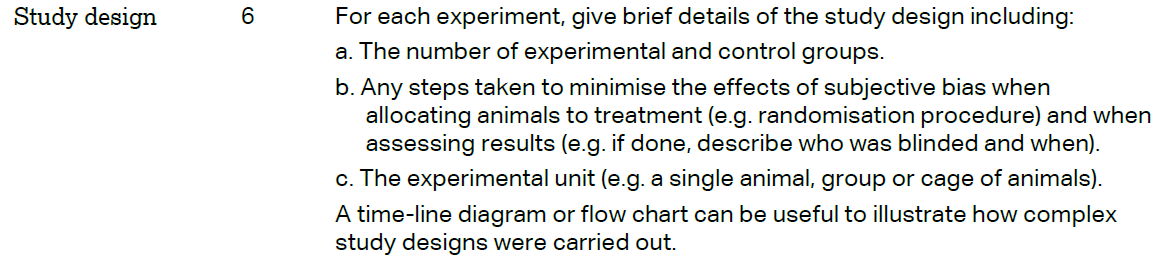 | | | Materials and Methods, In vivo validation, Paragrah #1 |  |
| 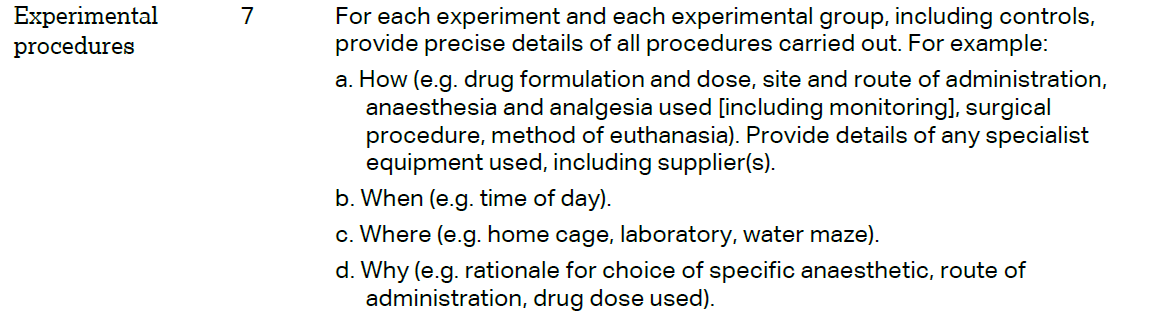 | | | Materials and Methods, In vivo validation, Paragrah #1 |  |
| 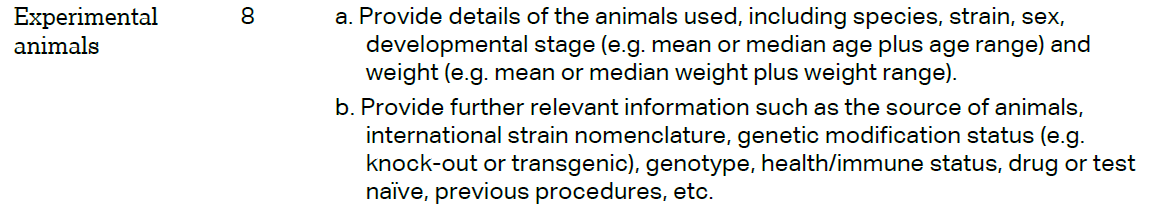 | | | Materials and Methods, In vivo validation, Paragrah #1 |  |

The ARRIVE guidelines. Originally published in *PLoS Biology*, June 2010^1^

| 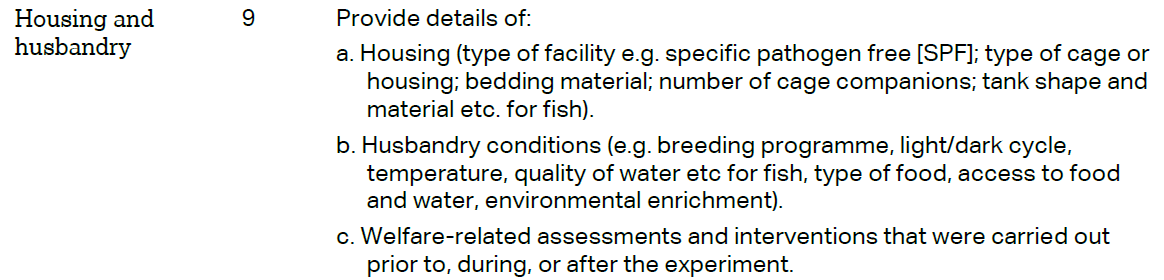 |  | |
| --- | --- | --- |
| 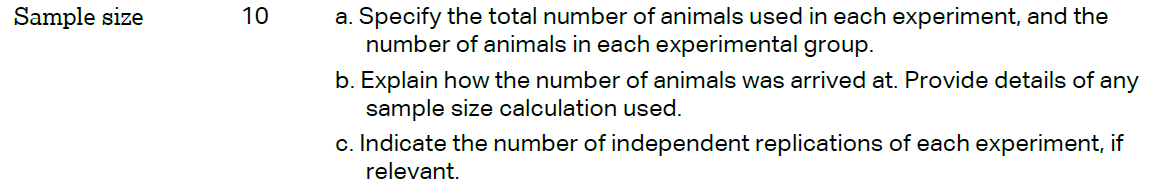 |  | |
| 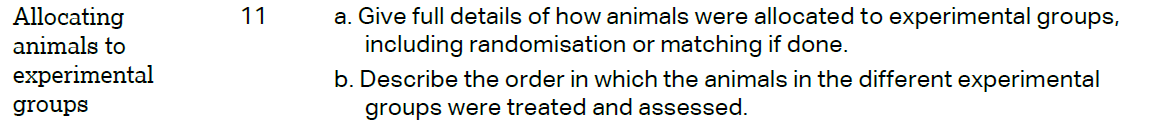 |  | |
| 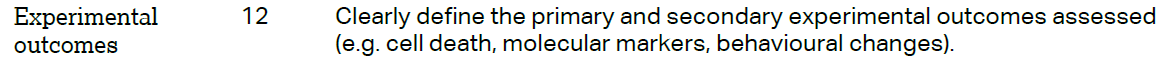 |  | |
| 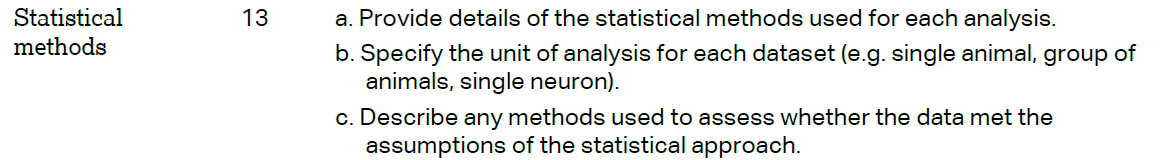 |  | |
| RESULTS |  | |
| 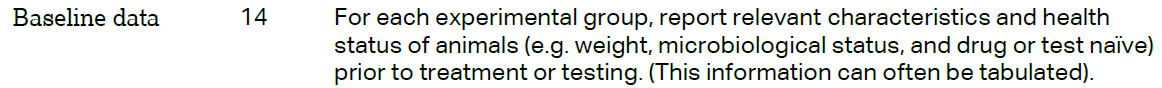 |  | |
| 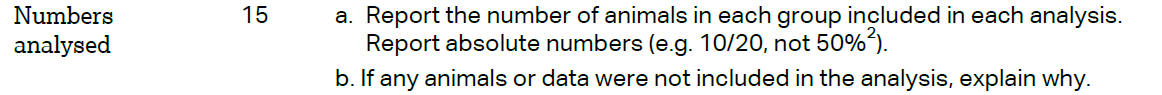 |  | |
| 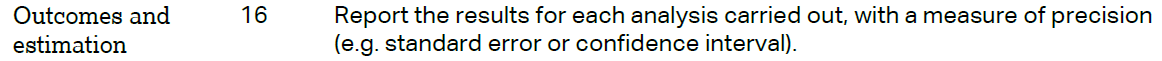 |  | |
| 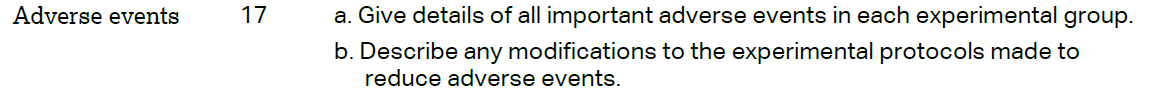 |  | |
| DISCUSSION |  | |
| 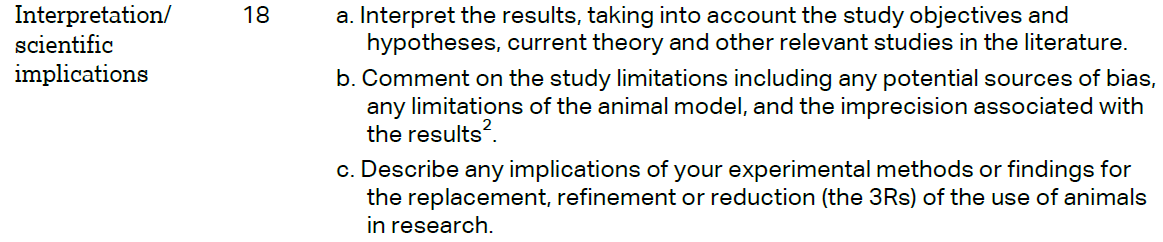 |  | |
| 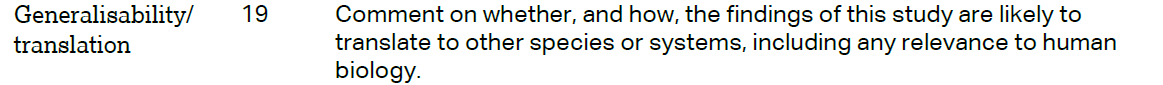 |  | |
| 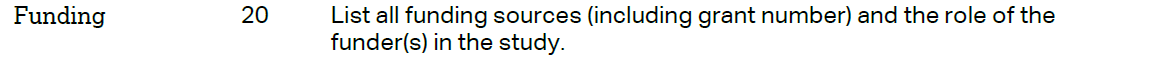 | |  |


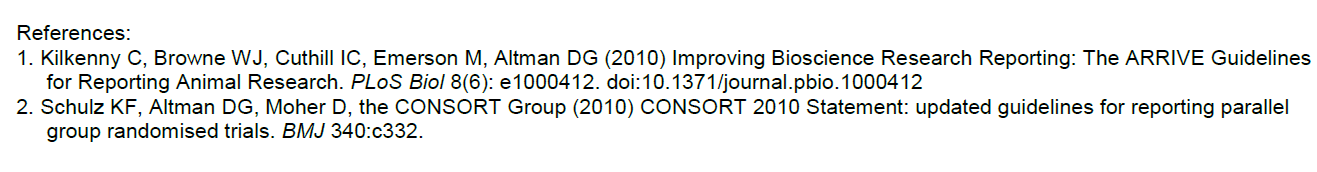

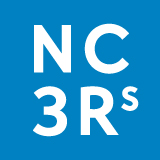

Supplement: S1 Arrive Checklist — (DOCX) [file pone.0124786.s001.docx]
