# Supplementary material for: Prediction of CD8+ Epitopes in Leishmania braziliensis Proteins Using EPIBOT: In Silico Search and In Vivo Validation
Source: PLoS One. 2015 Apr 23;10(4):e0124786. doi: 10.1371/journal.pone.0124786 (PMC4407964; doi:10.1371/journal.pone.0124786)
Supplement: S1 Table — (DOCX) [file pone.0124786.s002.docx]

Supplementary Material S1: List of IEDB CD8^+^ epitopes previously described used to train EPIBOT.

GTDSGFAAY

CTDDNALAY

LTDDMIAAY

KSDGTGTIY

ISDYDYYRY

LSDDAVVCY

CADGTRHTY

VVDKYFDCY

FTERSDKSY

FADINGKLY

TCDGNTFTY

LADTSLSGY

PVDEYITTY

NQDLNGNWY

EVDPIGHLY

SSLPTTMNY

EADPTGHSY

TQDLVQEKY

TSYVKVLEY

ATDFKFAMY

DSDGSFFLY

IADMGHLKY

STDHIPILY

YLDDPDLKY

YTAVVPLVY

YTSDYFISY

CTCGSSDLY

LSAFSLHSY

ASADKPYSY

CSNDKSLVY

CTELKLSDY

FLDQWWTEY

FRDYVDRFY

GTATYLPPY

GTQLFEDNY

HLDMLRHLY

HSAEALQKY

HTEFEGQVY

IIEDQYNRY

ISDESYRVY

KAEVHTFYY

LLDTASALY

LSGNGHFHY

LTAGLDFAY

LTCSPQPEY

LTEIDIRDY

LTKQYLNLY

LTYKYNQFY

NTFVQANLY

PTTGRTSLY

RLDAFRQTY

TSPYELSLY

VQDCNCSIY

VSDGGPNLY

KVSDEIWNY

LIENELMNY

NLALLYGEY

NVDQQNDMY

QAAESNERY

SSFFMNRFY

SSPLFNNFY

VSSIFISFY

ATDALMTGY

ATDALMTGF

VAEMDGIQY

CTLSEQLDY

YIESKRGVY

FMGRIRSVY

FIAQSKGLY

SSRMYCSFY

IMATIQRKY

LRSEAFEYY

LTGVEAVMY

VMYMGTLSY

MMSAPPAEY

GTFLCANEY

TTTIKPVSY

EIEPKLDGY

LSNFGAPSY

LAAEWVLAY

YMLFTKFFY

TSSTCMMCY

VSVKMFDAY

WFSQRGGSY

FSAVGNICY

YTPSKLIEY

GTDLEGKFY

LMNVITLVY

ITLVYKVYY

LVSTQEFRY

TDDNALAYY

ISRQRLTKY

LSFKELLVY

SSGDATTAY

DHEFVDEFY

LTGHMLDMY

LTNDNTSRY

NTLTLAVPY

DSKEGFFTY

HTSSMRGVY

TSSMRGVYY

CVADYSVLY

ASSEVAVLY

GAEHVDTSY

LGADSSIAY

KTSVDCNMY

LTALRLCAY

YSNRNRFLY

FTSCELYHY

VFHLYLQYI

FYTTTGIGY

TVAYFNMVY

AMDEFIQRY

SAAAYFVGY

ATSTGNYNY

STGNYNYKY

ATSRTLSYY

LSPRWYFYY

QSITRSLIY

FTIDFKLKY

VWINNSWKF

DMCDIYLLY

GTHVLLPFY

QSDTVFDYY

SQSDTVFDY

RVDFCGKGY

TQDLFLPFY

VLWAHGFEL

FVDGVPFVV

LSDHQDLKW

TSNPKTPKY

SSLPSYAAY

SSEQTFMYY

KSDPIMLLK

IVDCLTEMY

KGSGKMKTE

ALMEITSRY

KSDLQPPNY

IVDYVTAYG

MCFHQHLMY

GSEELRSLY

CSDDGFWSK

NSSKVSQNY

YFDPANGKF

CSEYVKDIY

KCDICTDEY

MIEPRTLQY

GTEMFRHGY

YINMAWNLV

MLEEMQSAV

YSDIPRLKK

ICDDVLSKY

GSDGGLDDY

SSSLTSLLK

YLDFGGPEG

ETEQPTLDY

SSECQGEML

ETFNTPAMY

YVDHYYRDY

GSEDRDLLY

YTYPCIPEY

YRSDIVGTY

FVSVYFSDY

LAEQFSGEY

VQLQEYDTY

MSDIFHALV

ALEPGFKDY

SYGNANVSF

SSMNSDAAY

YLGTPNNTY

HTAEIQQFF

RMMGKNIFY

YTDDYPMYK

HSSVAGGLW

FMGRLGPEY

WSFLEDRVY

QTSTLYDFY

STHMENILK

TTFPVNGGY

EVDEGSDMM

NSDPEFNVL

STDTRHIPQ

AHGWSTFYL

WTDLYTSMS

ISDSAQNMM

YTFCRLNVK

RSEVELCIY

DTDISQLHH

YFRNSGMTY

ATQPVHWFL

ISNYICVAW

PSSKPDWFY

ITAGYNRYY

MQLQLNCAY

QSSFYSDWY

DVAAMSGYY

FVARIDLGY

MTGVMRGNY

GTEKLTITY

ISDEFMWRY

LTDRELLLL

NSDEQSLEY

MTDKICWLY

YTDKIAMSY

LTDKSGSEY

LTDNGYLLY

LSDNLSLVY

ETESATLFT

SSDDFALIV

AVNPGGNIY

ISEKLRSGY

AADSFATSY

HSNLNDATY

MIDGIGRFY

NMDKAVKLY

WSQNPTMLY

NSDTVDWSW

LSDLKKTIY

VSFDQNLDY

ISEMLSKEY

KTDAGASTY

KTDIVNTTY

NTDAFSREY

VSEHFSLLF

MTDLSKKGY

FTENGPWMY

FTDISMSLY

NTDNKFISY

MTRVTNNVY

VSEKYTDMY

ITDFNIDTY

HAEQGLIQY

ATEDPSSGY

NMDRAVKLY

TTDDSTSYY

FSDLCNFLI

LSDAIFDDL

YSGNIVHRY

LSDLCNFLV

LTSSSKYTY

KACDLAMCY

FMSRKLHRY

HSDAVEDFL

HTVGLGQGY

FSLPSSSSY

ATATWFQYY

ALTSLGLLY

AIDLDPVVY

KSAAIDGEY

ETACLGKSY

RSNDTELNY

ESTINLLPY

ASSSNYNTY

SSHNHIPGY

DSDDWLNKY

LSDLPGLGY

ALTDLGLIY

YENAVWDQY

LIDGRTSFY

YSQESPQSY

YLSPFKLTY

CTLNKSHLY

HTEFQTVSF

VSDRPVMRY

ESSINISGY

ATDPVEMAL

KSSLEVYIY

EISGSSARY

MNNGGDAMY

GTGDSRLTY

GVEGIGLQY

NTDIKTLKF

AVTALTIAY

EVDQTKIQY

YANMWSLMY

HSEEGSRAY

DTACLAKSY

RSGIDTNAY

RTWNYHGSY

KGDTTTGVY

LTKEEFTRY

QTDNQLAVF

ITYTDVLRY

MTKEEFTRY

VVEKQSGLY

MTTTANWLW

SVEVKLPDY

LMSGKDVFY

YAQMWTLMY

YAQMWSLMY

ESDPEGALW

YTDLTYQSF

LSDAARLFL

QTYMYTGQY

LLDGLLAWY

PTTTYTLEY

LTDSDSPTY

FSIPVTFSY

RTNGASYAY

PTAIYTLEY

QVDQGIEYY

MTSGSSSGF

GTDSNGMLW

MLAPSTLGY

DTDPLPVVF

NIDPEHLDY

ITMVNSLTY

YQNEVTPEY

IQDEIVAAY

FTNKLINGY

DTAKPTSVY

WSDLNTTDF

MLSSFGWIY

WTALMFAAY

CTDKFSQLF

CSEVPQSGY

ATSGYRIAY

PSEKRIGAY

VSDTTVLLH

ETESVNSNY

CTDPYSQMV

YIDNTTSWY

NTAIFDMLY

FIYFGKKQY

YTFEPHYFY

ATNNLGFMY

NTTQQGDMY

FLMRNAIQY

KLEYLAPSY

WTIGNRAPY

FVNRYGVAY

IYDFYNAEY

RADSMMLGY

STYQPLPLY

YRTAVCGLY

NTSTCFQEY

HVDIPLQAY

RPRLHSISF

VPAERRGVF

RPRVAQLTF

LPAEVRAAF

YTVRGTGKY

LSSKNNEHY

PTDYAKPQY

KVQEWYLSY

PSDFFYLLF

FTDNNELEF

VTECKLIYY

FSFEIALLK

MSDIFASEV

SSEADCFTY

SSSGMDAYY

MTFPVSLEY

QSDIAGAIH

VTFFCVMTY

ILRPLGIEY

RSTLANGWY

HTAAPWGSY

NSSYWRQGY

ASEELMDKY

MLDQFGVSY

ETIEDYLGY

TTSDFFVNY

WSADGSSMY

KTDVIDLLY

LSSIGIPAY

YSDIFNNVL

LADVCNWTY

WTEHRQVRY

STFATVLEY

FSDVSHWWQ

LSDDSGLMV

LTDAFHGYH

TSEHGGRAY

TSASFTDLY

WSTIWRQLY

SVDSDHLGY

LVDENQSWY

GTEYRLTLY

RTDNGGWAH

VTSSVSSGY

NTAINFFLY

NTDDFPLTL

STNTLPTEY

WTDLFDNKV

VTDTALAYF

NSESGNSRY

NTFKFGVIY

SAEVVTLWY

CASSSDWFY

AGRAWENTI

AGSKYIHCF

CGDPSSFDY

CGDPSSFEY

CGDPSSLDY

CLERWMLVA

CSANNSHHY

CSKILDLCY

CSYKIGHHV

CTNFKTQLV

DCIMTSYQY

DHHFTPQII

DSEEYHLLY

DTGNYILCY

EALRGFLLY

ECSDSPLVL

ERSASGGVY

ESALNISGY

ESDGKPQKA

ESDGKPQKV

ESDSKPQKV

ESQRYIHCY

EYDFNKLLV

FLDGVNLVA

GIDVTDLFA

HCSQVFLKM

HTNFESFTV

ICSSVLKRY

ISEPTIHLV

ITDVTTLVV

ITEMLQKEY

ITEMLRKDY

KDFKCFNLI

KLSHSDYEY

KVNACHHNY

LCYALDLLY

LEIICFHEY

LFDVIPVSY

LFQFFVFLV

LINLTTIAY

LISIFLHLV

LITEQFLCY

LKILVLSIL

LSEISFHLV

LSEMLNKEY

LSSGEPHCA

LSSSEPHCA

LTEEFYHSY

LYDVIPVTY

MCPFLFLAV

MIDNQKLSY

MLDDLTMGY

PTDPVELAV

SCEEGKLCY

SDAHKKNLY

SSGCYIHFF

STGNYVHCF

TADDITMGY

TTDFTRLRY

TTDVKAAVI

VDICFWSTI

VGEEFFHQY

VPDIPELSY

VSDRPMMRY

VTDVTLLMA

VTERIFREY

WCEFVDFSV

WCSQTDYQY

WCSQTNYQY

YCNYTRFWY

YKELCDAVY

YSEESPTEY

YSEESPTSY

YVQRFFLRV

YVSSIFLHL

YSDPKRFFL

ASPILRFLY

GSTELSPLY

KTGESSRSY

KVNTTIARY

LTDNDDILM

NANAYSGKY

MTKEASREY

CMLNNSFYY

CSSLTEEFY

YNISRKIVY

HSIKRNYPY

STNTGNLKF

YTVKFPNLI

KSFKDQSKY

HYLCLNCLS

CRHCLNLLL

LFQLIFFLT

QSDTVFDHY

MTDVDLNYY

KVDWNQFTY

LTAHYCFLY

ASENSSAMV

ATDVPSATK

DSDNTQSEH

EAEKQLQQY

EIDTTIGEW

EYAPFARLL

FLESGAVKY

FSTSDGKEY

GSYGEYQSY

GTGPCPGDY

GTNETEYLF

HKDGAFFLY

HKEGAFFLY

ISDRATRKY

LFEVDNLTY

LLQLNETIY

LTESDMDYH

NSTHNTPVY

PDDGSYGEY

QSEHSFEEM

SLPKTSGHY

SSLAKHGEY

TEDPSSGYY

TENTSSYYA

TLDGQQFYW

TSDGKEYTY

TSSYYATSY

TVAPPAPVY

VTEIDQLVC

VTLDGQQFY

VVDPDDGSY

YTENTSSYY

AMEKSSKYY

ETDLRSEFD

FTEQAFYTR

GTEKEFLRY

GYCMIRWLG

ITCKAFGLY

ITDFHERPV

KLNGAMVEY

KMDNGTLEF

KSNAKCIEY

LSLTKLFSY

LTDSLSSQM

LYQLLEAVY

MLEGETKLY

PIDSHKGYV

PSIATNLEY

QIDKNKLYL

RGDLETLGY

RSDGYFLKI

RVNPGTYVY

SLFTEQAFY

VTAASPMLY

WAMEKSSKY

YVTLNASQY

YLEPAIAKY

DIEITCVYC

NTGLYNLLI

FKDLFVVYR

CIDFYSRIR

YNLLIRCLR

LQDIEITCV

ISEYRHYCY

ASAAHLAAY

ITDWLNFTL

QSAANMYIY

VSALRLFNY

KTAVVVTRY

FLQMENLMY

RTLDTLALY

TLDSEDGLY

YTTDVNQLY

FAADKDSLY

MTAAQIRRY

TTALVQCIY

TSADQQSLY

GSQYVSLAY

RINEGWPAY

MTRGLLGSY

FSDLLSMAW

MLASIDLKY

STIVWSSRY

CLLSHTLAY

ITDYIVGYY

FTAMQALDY

LTMDREMLY

VSTWQGFVY

HSETVIHRY

WTGMVDGWY

ATLTWVDWY

YSALRPHEY

VVDALRNIY

LSTASSWSY

MVASQLARY

SASLAALFY

ITDITKYLY

LANWCLLNY

MSPDNALIY

NTLGITHLY

FASSRMSTY

RTDAWSYPV

TTITGDSAY

FTWSGDVRY

ATTFARFLY

VTIGNAYIY

FVADSTPLY

HSNLNDTTY

ISANANFSY

YSSPHLLRY

YVSVVSSHY

FVMSCKLLY

ITSKEVFTY

LLDNSMFTY

ITTFFTFAY

RQIRMTSTI

GTFDLGGLY

MAAAGATLY

MVEASGGRY

GTTEVNGLY

VSFNQNLEY

AVASLLDEY

FTIRDVLAY

ASIADILTY

NRDGDSYYY

WVAGVQLLY

ETIEILRNY

FNCGGEFFY

GSEEIKSLY

KVFDKSLLY

QQYHRFGLY

YVYFYDLSY

ISDPLTSGL

IYTDEVYDY

LSMFVTNKK

SSNPVMSRF

SVDGFRASY

TFMDHVLRY

LVFLGPGLY

WMSNGTWNY

EIAQHGAWY

FTFWTFANY

GSASPTPPY

HSDTHGLYW

HSNASTLLY

RVFPGDHFY

YTIGIGAFY

LSEEANWAF

MSAIVSCRY

TGIAIIAYI

YLDNVGVHI

DTGCRIDGY

GTEELKSLY

MLYPRVWPY

MSSAAHLLY

NSDPNTPDK

TRAPAPFPL

TTRAVNMEV

EIYRTLYGL

FYHISTGGY

FYPINDDFY

KQIVIINPM

MMDLSHFLK

MYPFIFFIV

SWNNHSYLY

VSMDQLASY

WAIQCYTGV

ELAPIRVNA

FTWQHNYYL

NGNFNFERV

SVFELSNFA

TVFRNQNRV

EPFSRRHPL

ERAFQNWSV

EVVDMLSTY

FTLINWRSV

IESNPLFPV

NADTGHSIY

NPAACSYMV

YYKDDISYF

EGAGIDDPV

FFSPFFFSL

FMVYVPLPA

GTEEIKSLY

GTEEIRSLY

AVHGYYIGY

IIYYQLAGY

KLYPNVDFY

RLASYGLYY

VLDMGDPVK

YQAENSTAE

FGALFMWLL

GVNFNLYYY

KQIGGTLFE

NVMDPMHGA

QQRPDLILV

SSDLRSWTF

ATADLELAY

GVDGGWQAL

KMFHGGLRY

KPIPHRTVL

MGMEQTMSV

MVFGRFSFA

RVACRDVEV

ATFRLECPY

EGFDPRALI

HQFTSNPEV

LTFLDCLYY

MTACGRIVV

EDFEIFYNL

ETVWPFFYA

FTARIIIFS

FVMPIFEQI

KSNRIPFLY

MTFPLHFRS

MTYLDGHPV

TQSPVSVGF

ESENISEPY

EVIRATYPS

EVRKAIEFV

FVAAFDHFY

HMMAVTLFY

KYAEAFQMV

QVIFKCVPK

SQYDPKELL

WDAYIPHYV

WLYDLWGQL

ALDISFTGA

AALEGLSGF

GSDCTTIHY

IVDSLTEMY

KSCLPACVY

YSHGTGTGY

MSQIMYNYP

AMEDLVRAY

AMMARDTAE

MMARDTAEA

NATDTWVTY

TSEIQLTDY

LTDYGALTL

GTVLVQVKY

LTSREVLLL

VTRGAVLMY

KTKNNDWDY

YSDRRWCFD

YSDPLALRE

NSEQGGKAY

LSKSEFNTY

ATSIFKLTY

YAQMWQLMY

VSSWEEVPY

VVQPENLEY

ITEAELTGY

QMSSGNLLF

STSLSVSLV

QIDNFSLGV

MTDDIGMGV

LTDALALGM

YSQIGAGVY

KVDAIDGEY

ITASILLWY

VIDLDPIPY

YTDYMPSMK

NLTSTWVTY

TTEAILPEY

KLELKGMSY

GTILIKVEY

YMGEDGCWY

ASDRMGMGV

LTSRENLLL

KTDWLPMTV

YSIPATLLV

VTRGAVLTY

YTDRKWCFD

STANVSLAA

TIDLDPVIY

LSRKEFDLY

VTTWEDVPY

VTYKCPLLV

NLTSTWVMY

LVQIENLEY

DTSEVHWNY

GTTVVKVKY

ISSTPFAEY

NSALTLHWF

FLGEDGCWY

LTSRETALM

ETNMITLLV

LSSTRVPNY

FTDPSSVAA

PSSVAARGY

LTDWDFVVT

LTEIASLPT

TTERGGRAY

MTAGIFLFF

KTDFGFYQV

NTSANLSLA

LTASLVMLL

VIDLEPISY

RTWAYHGSY

NSEQGGRAY

VTARWLWGF

IVQPENLEY

MTTEDMLAV

VVQHENLKY

FTNMEAQLV

VTTWENVPY

VTYECPLLV

FIDGISLGL

TTERGGKAY

FTTNIWLKL

FTDPASIAA

TTDISEMGA

WTEAKMLLD

YSQVNPLTL

KTWAYHGSY

MTDTTPFGQ

FTNMEAQLI

YSDPLALKE

LSDSTMTNY

ISDYFHNTY

NIDTALTLY

VSDDSQNDY

NVDEQSGLY

NVDIFNPKY

FTGLIGMLY

NLENAAELY

YLEKESIYY

CTTYGTDLY

KIEEQSGLY

LTYYKLNTY

VSVGNTLYY

ATNNIHWSY

STEGSTLYF

LSALRTGWY

FTPNEPGKY

FLEQKVIKY

LSVLRTGWY

VSNWFSNWY

KIAACNDFY

NTAKSNQLY

YAAEIGIQY

ITDMRPLSL

ALHNLVLSY
