# Supplementary material for: Prediction of CD8+ Epitopes in Leishmania braziliensis Proteins Using EPIBOT: In Silico Search and In Vivo Validation
Source: PLoS One. 2015 Apr 23;10(4):e0124786. doi: 10.1371/journal.pone.0124786 (PMC4407964; doi:10.1371/journal.pone.0124786)
Supplement: S2 Table — (DOCX) [file pone.0124786.s003.docx]

**Supplementary Material S2: Proteins with known epitopes used to train EPIBOT.**

CAA46472.1

CAA33899.1

P19390.1

P14635.1

P17392.1

P00403.1

P11926.2

P17395.1

P13639.4

P03375.1

P04626.1

P04308.1

P14921.1

P03347.3

P12494.3

P04594.3

P01861.1

P04722.1

P04724.1

P28730.1

P09790.1

P16913.1

P04298.1

P04318.1

P14239.1

P04935.1

P13699.1

P09992.1

P07400.1

P26578.1

P12004.1

P07564.2

P27915.1

P26664.3

P26663.3

P26662.3

P03314.1

P29165.1

P14240.1

P14241.1

P20532.1

P21004.1

P24770.1

P03296.1

P21103.1

P04305.1

P03126.1

P06463.1

P08669.1

P09991.1

P18141.1

P12927.2

P18072.1

P18541.3

P30279.1

P29991.1

P29846.3

AAB81004.1

CAA48977.1

AAA43467.1

AAA43637.1

AAA48058.1

P14679.3

P33051.1

CAA52275.1

P33478.1

P35262.1

P34014.1

P33861.1

CAA50404.1

AAA64785.1

AAA46259.1

AAB60340.1

AAA82176.1

CAA88930.1

AAA69166.1

AAC13780.1

AAC54695.1

P46082.1

P43355.1

P43356.1

P43357.1

P43929.1

P43937.1

P44003.1

P48242.1

P16713.2

CAA67498.1

AAC55091.1

AAC55092.1

P47597.1

P52356.1

AAB39353.1

CAA73940.1

P51041.2

CAA76165.1

AAC14894.1

AAC14895.1

AAC14896.1

AAC14898.1

AAC14902.1

AAC14904.1

AAC14905.1

AAD03396.1

CAB37419.1

AAD34096.1

AAD51926.1

BAA83653.1

O68006.1

AAF18105.1

AAF18112.1

AAF18113.1

AAF32126.1

O05087.1

O83639.1

Q66802.1

O11459.1

Q05318.2

AAF75994.1

AAF81516.1

AAF86704.1

AAF86710.1

AAF86711.1

NP_059433.1

NP_041726.1

BAB12562.1

Q9YGY0.1

NP_005137.1

NP_052637.1

NP_053122.1

NP_058218.1

CAC17504.1

NP_041724.2

AAF20092.2

BAB19174.1

P26358.2

AAG58600.1

NP_076407.1

BAB41645.1

BAB43405.1

AAK55078.1

AAK60497.1

AAK60498.1

AAL01685.1

AAL12709.1

CAD00564.1

CAD05465.1

CAD01893.1

CAD02061.1

CAD05580.1

CAD02487.1

CAD02502.1

CAD07659.1

CAD02692.1

CAD05850.1

CAD05867.1

CAD06064.1

CAD02838.1

CAD06877.1

AAL52105.1

AAL53555.1

NP_601923.1

BAB88071.1

BAB88075.1

BAB88077.1

BAB88540.1

CAC84754.1

Q9HTE9.1

Q92J75.1

BAC04181.1

AAL59738.1

Q59599.2

BAC15762.1

AAN09938.1

AAN05424.1

AAN37504.1

Q13425.1

Q8RQL6.1

BAC28319.1

AAO07968.1

AAO10081.1

CAD70678.1

AAO90367.1

AAO90371.1

AAO89292.1

AAO89328.1

AAO89490.1

AAP17274.1

P59595.1

P59632.1

P59633.1

P59635.1

P59594.1

P59596.1

AAP22088.1

Q06210.3

Q87L45.1

AAP79751.1

AAP79852.1

AAP88031.1

Q87MX4.2

AAQ55254.1

AAQ55263.1

Q9UQD0.1

P29144.4

BAC93293.1

BAC97625.1

Q9H1A4.1

AAO59509.1

AAP44552.1

AAO59514.1

AAO59515.1

P06935.2

AAR17902.1

NP_942124.1

BAD02346.1

P09866.2

AAS22102.1

AAS77635.1

AAS77645.1

AAS77646.1

AAS77879.1

EAK87991.1

EAK89133.1

EAK89397.1

AAT05469.1

EAL12477.1

EAL14458.1

EAL15607.1

AAT40446.1

AAT40454.1

AAT40455.1

AAT40456.1

AAT49006.1

AAT58050.1

P26313.2

AAU49262.1

P63261.1

P62250.2

P67125.1

P65003.1

AAV53168.1

Q9JFA8.1

Q775Q3.1

Q9JF79.1

P68593.1

EAL47247.1

EAL49146.1

EAL50898.1

EAL50926.1

AAW31411.1

EAL56425.1

AAW57872.1

AAX19004.1

P0A558.1

P0A5Q5.1

P0A5G3.1

YP_232947.1

YP_233093.1

Q8X8F2.2

P02863.2

AAY82055.1

AAY98604.1

Q9WMX2.3

Q57JL7.1

Q87KE1.1

Q57DB3.1

Q6MWW4.1

Q6MWY2.1

O53767.1

Q58HT7.1

Q5UB51.1

Q5XX03.1

Q6J3P1.1

Q8B115.1

Q91DD4.1

Q91DD5.1

Q91DD7.1

Q9IQ47.1

Q9WDA6.1

Q6YMS3.1

Q6YMS4.1

Q6DV88.1

Q8QQ34.1

Q3I5J3.1

Q3I5J2.1

Q3I5J0.1

P0AE17.1

ABD19536.1

CAJ01212.1

Q9NQW6.2

CAJ20722.1

ABF80138.1

ABG16349.1

ABG16624.1

ABG16845.1

ABG17349.1

ABG17446.1

ABG17464.1

ABG17976.1

ABG18507.1

ABG18522.1

ABG19245.1

ABG19787.1

ABG20235.1

ABG13255.1

ABG13258.1

ABG42502.1

ABG43211.1

ABG43317.1

ABG43575.1

Q1RHX0.1

ABG67747.1

ABG68311.1

ABG68757.1

ABG69490.1

ABF04835.1

O95620.2

CAL34268.1

CAL22320.1

NP_033507.2

ABJ01631.1

ABJ55415.1

Q8N461.2

P27909.2

P17763.2

EAW86629.1

EAW91607.1

BAF42671.1

ABL96585.1

ABM49342.1

ABM49996.1

ABM50450.1

CAL71509.1

CAL71543.1

CAL71904.1

CAL73320.1

EAX58236.1

Q8BUR4.3

Q2A5M8.1

Q1RJ74.1

EAY58445.1

BAF48755.1

CAL29860.1

EBA41824.1

EBA42384.1

P13647.3

AAW51410.2

ABQ60400.1

EDM60245.1

ABS45863.1

ABV16863.1

EDO76704.1

EDO77069.1

EDO77377.1

EDO78537.1

EDO78551.1

EDO80494.1

EDO82575.1

ABV75419.1

ABV79247.1

P08603.4

P03431.2

NP_056776.2

ABX61793.1

ABX69443.1

EDR16741.1

EDR16869.1

EDR17131.1

EDR18282.1

EDR19727.1

EDR20473.1

EDR22270.1

EDR22496.1

EDR89884.1

EAL49829.2

EAL49579.2

ACA96816.1

ACB02469.1

ACD08643.1

AAI36952.1

ACD65199.1

EDV68565.1

BAG63735.1

EDX32452.1

ACJ70112.1

ACJ70113.1

ACJ70115.1

ACJ70116.1

ACJ70120.1

3D08

ACP15701.1

ACS92692.1

CAY54134.1

AEK26889.1

AEK26893.1
